# Inpatients’ awareness of admission reasons and management plans of their clinical conditions at a tertiary hospital in South Africa

**DOI:** 10.1186/s12913-015-0754-y

**Published:** 2015-03-06

**Authors:** Langalibalele H Mabuza, Olufemi B Omole, Indiran Govender, John V Ndimande, Herman S Schoeman

**Affiliations:** Department of Family Medicine and Primary Health Care, Sefako Makgatho Health Sciences University [formerly known as University of Limpopo (Medunsa Campus)], Pretoria, South Africa; Department of Family Medicine, Faculty of Health Sciences, University of the Witwatersrand, 10th Floor Medical School, York Street, Parktown, 2193 South Africa; Department of Statistics, Sefako Makgatho Health Sciences University [formerly known as University of Limpopo (Medunsa Campus)], P.O. Box 215, Pretoria, 0204 South Africa

**Keywords:** Inpatients, Global awareness, Health care practitioner, Management plan, Practitioner-patient communication

## Abstract

**Background:**

Inpatient awareness of the reason for their admission and the planned management enhances patient compliance and empowers patients to be resourceful in subsequent consultations. The objective of this study was to determine patients’ awareness of their clinical conditions while admitted to an academic hospital.

**Methods:**

A survey was conducted at Dr George Mukhari Academic Hospital in Pretoria, from 6 to 17 December 2010, on 264 inpatients drawn from a population of 837 through a systematic sampling method. Data on inpatient awareness were collected using a researcher-administered questionnaire, which was available in English, as well as isiZulu and Setswana. Components of patients’ global awareness were clinical diagnosis, necessity for admission, planned management, possible condition cause(s), duration of admission, and planned investigations, operations and procedures. We conducted regression analysis on possible predictors of global awareness: age, marital status, occupation and educational level. The SAS (Release 9.2) was used for data analysis.

**Results:**

One hundred and thirty-six inpatients (51.5%) had global awareness of their clinical conditions and management plans. High degrees of awareness were reported on clinical diagnosis 206 (78.0%), reason for admission 203 (76.9%), planned management 206 (78.0%), and current medication 222 (84.1%). Fifty (18.9%) respondents were aware of their estimated admission duration. Patients who were informed of admission duration were likely to be informed of their planned management (p < 0.01). When health care practitioners did not volunteer information, most respondents (>69%) did not seek information. When information was provided, the majority of respondents (>70%) reported understanding the information. The proportion of patients who acknowledged the shared responsibility by the health care practitioner and the patient to raise awareness among the inpatients was significantly more than those who did not (p = 0.03). Patients’ age, marital status, occupation and educational level were not predictors of global awareness (p > 0.05).

**Conclusions:**

The proportions of respondents who were aware of the different aspects of health care ranged from 18.9% to 84.1%. About half of respondents had global awareness of their admission reasons and management plans. Raising awareness of patients’ clinical conditions should be part of the health care practitioner-patient encounter.

## Background

According to the European Charter of Patients’ Rights, “[e]very individual has the right to access to all kind of information regarding their state of health” [1]. Countries worldwide have adopted patients’ rights charters which seek to address patients’ comprehensive health care, including their awareness of their state of health [2-5]. Although patients’ rights to access information have been outlined in each charter, the process towards the realisation of this ideal is left in the care of each institution. There are two sides to raising patient awareness: the health care practitioner has the responsibility to inform, and the patient also has the responsibility to request to be informed.

Ideally, any patient whose clinical condition warrants admission to a health care institution needs to be made aware of the clinician’s working diagnosis, the reason for the decision to admit, and the inherent risks for non-admission. Once in the ward, the inpatient needs to be constantly updated on the management plan, including the estimated length of hospital stay, investigative procedures, medication and operative procedures envisaged.

At the time of the research, there was a paucity of literature in Africa on raising global awareness among inpatients about their clinical conditions and management plans. Studies that were conducted on raising patients’ awareness were on specific illnesses. A study conducted among women with breast cancer at the general surgery outpatient clinics of Lagos State University Teaching Hospital in Nigeria, identified patient ignorance on the nature of their illness as a risk factor for late presentation [6]. In South Africa, a study conducted at the University of Cape Town on pregnant women undergoing emergency diagnostic radiation found that only 7% of them had been informed of possible radiation risks [7], and another conducted in Gauteng on patients’ awareness of ototoxicity with MDR-TB treatment reported that only 20% were aware that their treatment had ototoxic adverse effects [8]. The Medical Law of South Africa states that “Many South Africans are not aware of their right to proper health care in this country. Everyone has the right to be given full and accurate information about the nature of one’s illness, for one to make a decision that affects one’s health” [9]. The authors’ literature search at the time of the study indicated that there were no studies conducted specifically on health literacy in South Africa. However, compared to Western countries, the continent of Africa has been shown to have a lower functional literary, resulting in lower health literacy – especially among women [10].

It has been shown that raising awareness on the patient’s condition improves patient cooperation with health care practitioners, and enables them to play a more active role in their own health [11,12], and also guides the patient towards realistic expectations from the health care team [13]. Contrary to this, patients who are poorly communicated with by their health care practitioners have been found to have a 19% higher risk of non-adherence to treatment, compared to those who were communicated with [14]. Furthermore, a systematic review on hypertension awareness, treatment and control in Africa found that on the whole, the African regions with higher awareness rates (North African countries) showed better hypertension control rates, compared to those with low awareness rates (East African countries) [15]. This implies that raising patient awareness has a positive influence on patient care. It is hoped that the results of this study will guide policy towards raising patients’ awareness on their clinical conditions in public hospitals in order to improve the quality of inpatient health care.

## Methods

A cross-sectional study was conducted at the Dr George Mukhari Academic Hospital (DGMAH) (formerly known as Ga-Rankuwa Hospital) from 6–17 December 2010. The hospital is the second largest hospital in South Africa; an academic hospital situated 30 km north of the capital city Pretoria, in the Gauteng Province [16]. It caters mainly for secondary and tertiary health care patients. Primary health care (level one) patients under the care of the Department of Family Medicine and Primary Health Care are also accommodated in the hospital since the nearest district hospital (catering for level one patients) is located about 15 km away from the GaRankuwa township which borders the academic hospital. It is a 1550-bed hospital with an average daily bed occupancy rate of 65.5%. It comprises 39 wards clustered according to clinical disciplines. It is the training hospital for health sciences students of the University of Limpopo (Medunsa Campus).

The study population comprised all inpatients at the time of the study. Twelve wards comprised the following: paediatrics (seven), psychiatry (two), kangaroo mothers (one), labour ward (one) as well as the burns and intensive care ward (one). Critically ill patients, patients younger than 18 years, and emergencies or mentally unstable patients were excluded because of ethical issues of consent. The remaining 27 wards (two level one, four surgical, four medical, one urology, three orthopaedics, two neurosurgery, one hand surgery, one ophthalmology, one cardiothoracic, one otorhinolaryngology, two post-delivery, one ante-natal, one gynaecology, one female oncology and one female burns), each had an average of 31 patients, resulting in a study population of 837. Using a 95% confidence level and a confidence interval of 0.05, the sample size was 264. With this sample size, any estimate of a population percentage (e.g. in this case, the percentage of patients with global awareness) will, with 95% probability, be within ± 5% of the percentage calculated from the sample, i.e. the estimate will not deviate by more than 5% from the percentage calculated from the sample.

Since the sample size represented 32% (264/837) of the total study population, the number of patients from each ward was calculated *pro rata* using this percentage. For example, in a ward consisting of 26 patients, eight patients were selected by a systematic sampling method. In this case, every third patient from the ward register was selected, beginning with a patient decided on randomly by the throw of a dice. This method was applied in all the wards. In the case where a selected patient declined participation, or was clinically or mentally unstable, they were excluded and the next patient in the ward register was requested to participate. Inpatients included in the survey were those who had been admitted for at least 24 hours so as to ensure a reasonable time of interaction with the health care practitioners in the ward.

A questionnaire developed *de novo* by the researchers, was subjected to peer review by an independent researcher and statistician, and piloted in a nearby 158-bedded hospital. The questionnaire was translated from English into Setswana and isiZulu (the predominant languages spoken at the research setting). It was then translated back into English to ensure accuracy of the translation. It was administered by research assistants (who were trained to ensure standardisation) to obtain information from every consenting patient. To minimise the potential bias associated with a patient giving a favourable or socially desirable response to the survey, the research assistants who were employed to collect data were not members of the hospital health care team.

The questionnaire collected patients’ demographic data and awareness of: the patient’s clinical condition, duration of admission, planned management, current treatment, investigations and surgical procedures (where applicable). It also obtained data on whether the patient had sought information and clarity on information given by the health care providers, and the level of the patient’s understanding of the information given. The level of patients’ understanding was measured using a Likert scale (not at all; somewhat; uncertain; fairly well, and fully). The questionnaire also enquired whether the patients regarded it their responsibility to acquire awareness about their condition and its management, and also whether the patients regarded it the health care professional’s responsibility to provide such awareness to patients.

Although our study bore similarities to a national survey of hospital patients conducted in England in 1994 [17], the components of their instrument differed from ours in certain respects, e.g. pain management, discharge planning and degree of patient satisfaction. Furthermore, patients recruited for their survey had recently been discharged from the hospital, whereas our study was on inpatients. Another national survey conducted in the USA used the telephone data collection method to investigate the extent to which perceived quality of care by patients was related to their characteristics [18].

Data from the questionnaires was captured using Microsoft Excel 2010 and was subsequently imported into Statistical Analysis System (SAS) (Release 9.2) software for data analysis by a statistician. Descriptive statistics were done to describe patients’ characteristics, and the proportion of patients who were aware of the reasons for admission and the planned management. A patient’s “global awareness” of the reasons for his/her admission and planned management was defined as the patient’s acknowledgement of having been given information on, and having understood all three the following components:

- What the patient was suffering from (clinical diagnosis),
- What necessitated the admission, and
- What the planned management was, plus**,** at least one of the following:
- Possible cause(s) of the condition
- Estimated duration of admission
- Treatment (medication)
- Specific planned investigations
- Specific planned and/or executed operation(s) and procedure(s)

For purposes of this study, the research team reached a consensus agreement on the components of global awareness as listed above. The first three on the list were regarded as fundamental in inpatients’ awareness, while the remaining five, though also important, were not regarded as fundamental.

“Planned management” was used as an overarching term referring to the comprehensive management plan in general terms, e.g. a clinician would say to a patient, “Since we think you have a chest infection, your sputum will be taken for investigations and you will be sent for chest imaging”. “Treatment” referred specifically to medication the inpatient was receiving. “Investigations” entailed side-room and laboratory tests as well as other special investigations (e.g. electrocardiogram, magnetic image resonance, etc.). Hence, “planned management” formed one of the components of global awareness, whereas “treatment”, “investigations” and “operations/procedures” did not.

Group comparisons were done using the Chi-square, *T*-test and Fisher exact tests where appropriate. These groups comprised proportions of patients who indicated their level of awareness according to the Likert scale. A logistic regression model was created to determine the socio-demographic variables which predicted patients’ global awareness as defined above. Statistical significance was set at p < 0.05.

The Medunsa Research Ethics Committee (MREC) of the University of Limpopo gave ethics approval for the study (MCREC/M/24/2008: IR). In addition, Chief Executive Officer (CEO) of the DGMAH gave permission to conduct the study. Written informed consent for participation in the study was obtained from each participant. Ethical principles of confidentiality, justice and autonomy were ensured throughout the study. Patient identifiers were excluded from the questionnaires to ensure anonymity.

## Results

All 264 patients who were recruited for the study consented to participate and completed the questionnaires.

### Respondents’ characteristics

The majority of the respondents (175) were females (66.3%), and the age-group mostly represented (54.5%) were young adults aged 21–40 years. Single patients formed the largest group (59.1%). One in two patients was unemployed (50.4%). Most patients (59.8%) had secondary level education (Table 1).

**Table 1:** **Distribution of participants’ baseline characteristics**

**Variable**	**n**	**%**
**Gender**		
Males	89	33.7
Females	175	66.3
Total	264	100.0
**Age group (years)**		
≤20	26	9.8
21-30	79	29.9
31-40	65	24.6
41-50	30	11.4
51-60	16	6.1
>60	47	17.8
No response	1	0.4
Total	264	100.0
**Marital status**		
Single	156	59.1
Married	72	27.3
Separated/divorced	7	2.7
Widowed	26	9.8
No response	3	1.1
Total	264	100.0
**Formal education**		
None	9	3.4
Primary	40	15.2
Secondary	158	59.8
Tertiary	43	16.3
No response	14	5.3
Total	264	100.0
**Employment status**		
Employed	76	28.8
Unemployed	133	50.4
Pensioner	39	14.8
No response	16	6.0
Total	264	100.0

### Patients’ awareness of their clinical conditions, necessity for admission, possible causes and estimated admission duration

**Figure 1:**
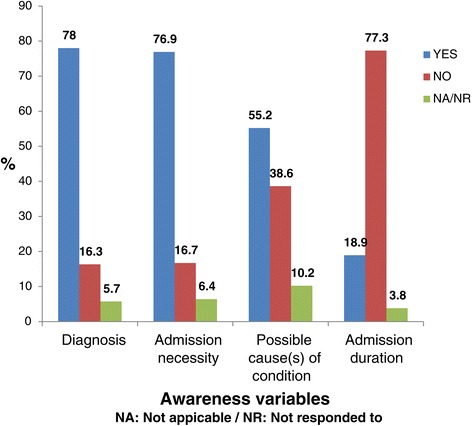
**Proportions of respondents with awareness on the clinical condition.**

Figure 1 illustrates the patients’ awareness of their clinical conditions, reason for admission, possible causes and estimated admission duration. It revealed that 78% of patients were aware of their clinical assessment/diagnosis and 76.9% were aware of reasons for their admission. More than half (55.2%) were aware of the possible causes for their clinical conditions, and the majority (77.3%) were not aware of the possible duration of their admission.

### Awareness on planned management, treatment, investigations and operations and procedures

**Figure 2:**
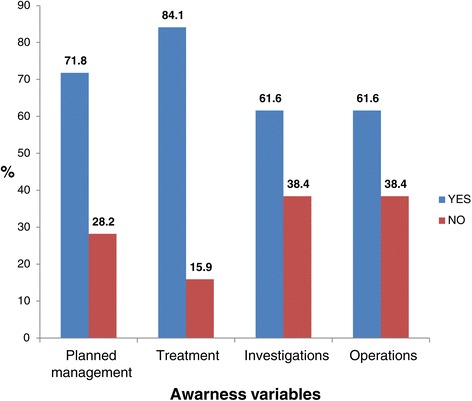
**Proportions of respondents with awareness on comprehensive management.**

Figure 2 illustrates that the planned management and treatment categories demonstrated a higher proportion of differences between those who were aware and those who were not aware (71.8% versus 28.2%; and 84.1% versus 15.9%), compared to the investigations, and operations and procedures (61.6% versus 38.4% in both cases).

### Seeking clarity on the various aspects of health care

**Figure 3:**
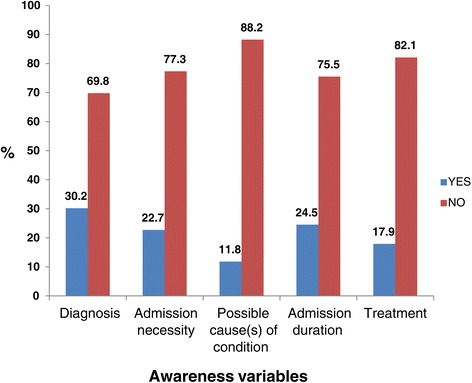
**Proportions of patients seeking clarity on the various aspects of health care.**

Figure 3 illustrates that there were low proportions of patients who sought clarity on the various aspects of their health care. This patient behaviour was most pronounced in seeking information for possible causes of their conditions (11.8%) and information on their treatment (17.9%).

### Global awareness and predictors of global awareness

Table 2 illustrates that 51.5% of the respondents demonstrated global awareness of the different aspects of health care as defined in the methods section.

**Table 2:** **Respondents’ global awareness on all aspects of health care**

**Global awareness**	**Frequency**	**%**
No	128	48.5
Yes	136	51.5
Total	264	100

A logistic regression analysis was performed with global awareness (yes/no) as a dependent variable, and age, marital status, educational level and employment status as predictor variables. The latter were not found to be statistically significant for global awareness (Table 3). On enquiry on whose responsibility it was to drive the awareness process, patients reported that awareness about their clinical condition and management plan was as much the health care professionals’ responsibility as it was theirs (OR 4.88, 95% CI 1.34 – 17.79, p = 0.03).

**Table 3:** **Logistic regression of global awareness and baseline characteristics**

**Variable**	**OR, 95% CI**	**p-value**
Age (≤40 versus > 40 years)	1.18 (0.67 – 2.09)	0.57
Marital status	0.96 (0.53 – 1.73)	0.89
Educational level	1.13 (0.30 – 4.36)	0.33
Employment status	0.79 (0.44 – 1.39)	0.40

## Discussion

This study investigated the awareness of inpatients about the reasons for their admission and the management plans of their clinical conditions. As far as the authors could ascertain, this was the first study in a health care institution in South Africa to document global awareness of health care among patients, which comprised: awareness of the clinical diagnosis, reasons for admission, planned management, and possible causes of the condition that necessitated admission, estimated duration of admission, planned investigations, and operative procedures done. This study found that only one in two patients reported global awareness of the different aspects of health care provided to them (Table 2), leaving almost half of all patients being managed at DGMAH oblivious to different aspects of the health care they were receiving.

Although the proportions of respondents who reported awareness on their clinical diagnosis and the reason for admission were high, at 78% and 76.9%, respectively (Figure 1), only 51.5% of respondents reported global awareness (Table 2), suggesting that awareness of other aspects of health care provided were low. The fact that 55% of patients indicated that they were made aware of the possible causes underlying the diagnoses indicated that some of the health care practitioners made the effort to provide the explanation. Giving a patient the possible underlying cause for a condition is important as it could clarify the disease mechanism and enhance patient adherence to lifestyle modifications and medication, e.g. a patient with mild hepatic encephalopathy with underlying liver cirrhosis from chronic alcohol abuse [19], made aware of the contribution of alcohol as the cause of the disease, could lead to modification of the patient’s social behaviour according to the Health Belief Model by Hochbaum, Rosenstock and Kegels [20].

More than 77% of the respondents were unaware of the estimated duration of their hospital stay (Figure 1). Awareness of the estimated duration of hospital stay empowers the patient and family for proper planning, especially for the self-employed patient who may not generate income while in admission. Furthermore, hospitalisation does not only affect the index patient. The schedule and planning of the other family members become impacted and may need adjustment during the patient’s admission. Hence, awareness of the patient’s possible duration of hospital stay becomes an advantage to the entire household. Interestingly, a high proportion of the patients (75.5%) did not request to be informed of the possible duration of their hospital stay if the health care professional did not volunteer the information (Figure 3). Studies have shown that patients are reluctant to seek information from their health care providers mainly to avoid troubling the health care providers [21,22]. This highlights the need to empower patients to ask questions about their health during a clinical encounter. About 72% of patients reported being informed of the planned management (Figure 2). Patient information on planned management has been found to enhance patient adherence [23], and preoperative information has been found to improve the surgical patients’ sense of empowerment, by reducing anxiety on invasive procedures [24].

It is noteworthy that, in our study, about 40% of the patients indicated that they were not given the opportunity to get clarification on operative procedures to be done (Figure 2). This raises an ethical question in patient care as each patient needs to be informed before signing the written informed consent for any procedure to be performed, including surgical interventions [25]. In South Africa, the main reason for doctors not to meet all legal requirements for informed consent has been found to be the notion by doctors that patients should be told by the doctors what is best for the patients [26].

Health care professionals often site time constraints for not providing enough information to their patients [27]. However, it has been shown that health care professionals can dedicate time for patient information within their schedule [28]. Lack of information is disempowering to patients [29]. According to the principles of patient-centred care, the health care practitioner’s busy schedule should not impact negatively on patient care [30]. Interestingly, this study found that where information was provided, the majority of respondents reported good understanding of the information provided.

Identification of predictors of a particular medical phenomenon affords health care practitioners the opportunity to focus on specific aspects in providing health care. This may facilitate timeous mobilisation of the required resources [31-34]. Our study found that a respondent’s age, marital status, occupation or educational level did not predict the respondent’s global awareness – similar to a study on antenatal care attendees in Ibadan, Nigeria, where no predictors were found for awareness on the cervical ripening and induction of labour [35]. This finding in our study suggests that in any patient encounter, the health care practitioner should not be guided by the patient’s baseline characteristics in raising global awareness about health care. Rather, it should be done regardless. Contrary to our study, Kahesa et al. found that the respondents’ demographic characteristics (age, marital status and educational status) influenced acceptance of cervical cancer screening among women living in Dar es Salaam, Tanzania [36]. This led the authors of that study to conclude, *inter alia,* that special attention should be paid to women of low education. This indicates that for certain conditions, health care practitioners may be guided by baseline characteristics in laying emphasis on dispensing appropriate health care.

A study has shown that not all patients want to participate in decision-making in the management of their conditions [37], and another that patients do not take an active role and enquire about the management of their conditions in due consideration of the busy schedule of the health care practitioners [22]. However, a study conducted in South Africa on reasons given by inpatients for not seeking clarity on what they had not understood about their conditions, indicated that patients actually wanted to be informed and involved in their management [38].

The acknowledgement by the respondents that the responsibility to seek awareness about their clinical conditions and management was as much the health care practitioner’s responsibility as it was the patient’s, came as a surprise finding to us, given that a large proportion of the respondents indicated that they did not seek information if it was not given voluntarily by the health care practitioner in attendance. This acknowledgement should be taken advantage of by health care practitioners to reach out to patients and raise health care awareness.

Global awareness on health is also determined by a patient’s health literacy, which has been defined as “the capacity to obtain, interpret and understand basic health information and services and the competence to use such information and services to enhance health” [39]. Improved health literacy has been linked to improved patient safety [40], while poor health literary has been associated with increased risk of hospitalisation [41,42]. In South Africa there are 11 official languages (English, Afrikaans, and 9 tribal languages), but most of the health materials available are in English [43] – a limiting factor in health literacy. Three measures have been found to improve health literacy: (1) making health literacy information more consumer-friendly (inter alia, cutting out the jargon), (2) helping health care practitioners to improve their communication with patients, and (3) supporting local communities in starting health education programmes [44]. The finding in our study that only about half of the patients displayed global awareness suggests the need for the implementation of these three measures in the institution.

### Study limitations

This study relied on information as reported by patients – with inherent subjectivity. Since patients were required retrospectively to report what they had been told on a specific health aspect, there could have been recall bias. To the extent that this study was conducted in a single health care institution, caution needs to be exercised in generalising these research findings to other contexts. The study did not enquire on the impact of varying levels of health literacy among patients – there could have been inpatients who had better awareness and understanding of their conditions as a result of chronicity, compared to those newly diagnosed or those with acute conditions. Furthermore, the study was conducted in a tertiary health care facility which predetermined the type of patients admitted as well as the level of care and interaction, and therefore does not discuss all the aspects of effective communication. The level of patient awareness may have varied according to ward speciality. However, our study investigated global awareness of all inpatients in the institution.

## Conclusions

This study has demonstrated that the proportions of inpatients that reported awareness varied, depending on the aspect of health care provided. Although the proportions of inpatients that reported awareness of some aspects of care were high (clinical diagnosis/assessment, reason for admission, planned management and current medication), only about half of the respondents reported global awareness of their admission reasons and management plans. Raising awareness of patients’ clinical conditions and management plans should be part of every health care practitioner-patient encounter. Future studies should focus on reasons given by inpatients for not seeking information about their clinical conditions and management plan.
